# Thromboprophylaxis with unilateral pneumatic device led to less edema and blood loss compared to enoxaparin after knee arthroplasty: randomized trial

**DOI:** 10.1186/s12891-022-05910-9

**Published:** 2022-11-15

**Authors:** João Alberto Ramos Maradei-Pereira, Marcel Lobato Sauma, Marco Kawamura Demange

**Affiliations:** 1 Faculdade de Medicina da Universidade Federal do Pará (UFPA), Instituto de Ciências Médicas, Av. Generalíssimo Deodoro, 01, Umarizal, PA 66050160 Belém, Brazil; 2 Hospital Maradei, Av. Nazaré 1203, Nazaré, Belém, PA Brazil; 3 Instituto de Ortopedia e Traumatologia, Hospital das Clínicas da Faculdade de Medicina da Universidade de São Paulo (HCFMUSP), Rua Ovídio Pires de Campos, 333, Cerqueira César, São Paulo, SP Brazil

**Keywords:** Hemorrhage, Edema, Knee Arthroplasty, Knee Replacement, Anticoagulants, Arthroplasty, Replacement, Knee, Embolism and Thrombosis, Venous Thromboembolism, Enoxaparin, Postoperative Hemorrhage, Intermittent Pneumatic Compression Devices

## Abstract

**Background:**

Pharmacological and mechanical thromboprophylaxis are frequently used together after total knee arthroplasty (TKA). Most studies in this context compare anticoagulants versus a combination of these drugs with an intermittent pneumatic compression device (IPCD). However, there is uncertainty about the need for the combination of both and whether a unilateral IPCD would alone affect other important clinical outcomes: edema and blood loss. We compared the effects of enoxaparin versus unilateral portable IPCD after TKA on edema and blood loss. We hypothesised that unilateral IPCD would cause the same level of edema and the same blood loss as enoxaparin.

**Methods:**

In this open, randomized trial (1:1), adults with no history of coagulation disorders, anticoagulant use, venous thromboembolism, liver or malignant diseases underwent TKA. For 10 days, participants received the IPCD, used 24 h/day on the operated leg from the end of surgery, or 40 mg of enoxaparin, starting 12 h after surgery. All underwent the same rehabilitation and were encouraged to walk on the same day of surgery. We measured edema (thigh, leg and ankle circumference) before and on the third postoperative day. Blood loss (volume accumulated in the suction drain and drop of hemoglobin and hematocrit in 48 h) was a secondary outcome.

**Results:**

We randomized 150 patients and lost 3 to follow-up with enoxaparin and 2 with IPCD. There was no case of symptomatic venous thromboembolism. Four patients needed transfusions (three receiving enoxaparin), one had infection and one hemarthrosis (both in the enoxaparin group). Leg circumference increased by approximately 2 cm for enoxaparin group and 1.5 cm in IPCD (*p* <  0.001). The increase in ankle circumference was about 1.5 cm in the enoxaparin group (*p* <  0.001), and almost zero in IPCD (*p* = 0.447). Enoxaparin group lost 566.1 ml (standard deviation, SD, 174.5) of blood in the first 48 h, versus 420.8 ml (SD 142.5) in the IPCD.

**Conclusions:**

Exclusively mechanical prophylaxis after TKA with portable IPCD only on the operated leg reduces leg and ankle swelling and post-operative blood loss compared to exclusively pharmacological prophylaxis with enoxaparin. Portable devices that can prevent deep vein thrombosis and pulmonary embolism without increasing blood loss or other risks should be further investigated.

**Trial registration:**

REBEC RBR-8k2vpx. Registration date: 06/04/2019.

**Supplementary Information:**

The online version contains supplementary material available at 10.1186/s12891-022-05910-9.

## Introduction

### Background

Venous thromboembolism (VTE), including deep vein thrombosis (DVT) and pulmonary embolism (PE), is a potentially lethal complication that can occur after total knee arthroplasty (TKA). This complication can be prevented using mechanical or pharmacological thromboprophylaxis or both [1, 2]. However, there is no consensus on the best choice. Currently, international research seeks a safe and effective solution to prevent VTE while avoiding excessive bleeding.

Postoperative edema greatly impacts rehabilitation, as it is associated with quadriceps weakness, reduced gait speed, and worse functional performance [3, 4]. In addition to causing asthenia and delaying rehabilitation, blood loss predisposes the need for blood transfusion, which, in turn, causes immunosuppression, favors infection and is also associated with a greater DVT risk [5–7]. Nevertheless, there are still few quality studies in the literature comparing the effects of the thromboprophylaxis method, specifically on edema and blood loss in TKA [8–12].

The American College of Chest Physicians (ACCP) recommends enoxaparin as a pharmaceutical choice for thromboprophylaxis [1]. Mechanical prophylaxis, generally recommended to be used bilaterally, is the portable intermittent pneumatic compression device (IPCD) [1, 2, 13–15]. However, its unilateral use has not been compared with pharmacological methods. Most studies on thromboprophylaxis in arthroplasty compare anticoagulants with a combination of these drugs and IPCD. But there is a gap in the literature for the comparison between isolated unilateral IPCD versus isolated enoxaparin regarding edema [8, 10, 12, 16–20].

### Rationale

As there is no standard for prophylaxis, and both IPCD and pharmacological approaches seem to work together, what should guide clinical choice for one or the other option, and are they really needed in combination? Is mechanical prophylaxis enough to prevent edema and blood loss in the context of TKA — even before any thrombotic event? And would a single-leg mechanical device safely provide the expected preventive effect?

To help with decision-making, we aimed to study the effect of isolated unilateral IPCD versus isolated enoxaparin on the edema of the operated thigh, leg and ankle after TKA. Secondarily, we compared these interventions regarding blood loss.

The hypothesis was that unilateral IPCD would cause the same level of edema and the same blood loss as enoxaparin.

## Materials and methods

### Trial design, ethics and reporting

This was an open, parallel, randomized equivalence trial with two arms. Adult patients undergoing primary total unilateral knee arthroplasty for any reason were randomized to one of two protocols of thromboprophylaxis: pharmacological (enoxaparin) or mechanical (unilateral portable intermittent pneumatic compression device, IPCD). The allocation ratio was 1:1.

The study was prospectively registered at REBEC on 06/04/2019 (RBR-8k2vpx), an International Clinical Trials Registry Platform, and it was approved by the public university’s institutional review board (Comitê de Ética da Universidade de São Paulo, protocol CAAE: 95636718.9.0000.0065). All methods followed the Declaration of Helsinki, and all patients received verbal and written explanations about the study aims and methods and signed informed consent forms for participation and publication. Patients were informed that they had the right to leave the trial any time. We report the study here according to the CONSORT 2010 reporting guideline [21].

After registration, we made two changes to the protocol. First, we inverted the primary and secondary outcomes: blood loss, which was the primary outcome initially, became the secondary, and edema became the primary outcome in this research. We then calculated a new sample size based on this decision, as described below. The second change was in the inclusion criteria: we decided not to include patients with body mass index (BMI) over 40 kg/m^2^, who would undergo more aggressive surgical trauma with more edema and bleeding. We also decided to include only patients undergoing unilateral surgery because blood loss would also be naturally larger in patients submitted to bilateral arthroplasty. Finally, patients in regular use of anticoagulants were excluded. These changes were added to the register.

### Participants, setting and location

We recruited for this trial patients 18 years old and over, submitted to unilateral total knee arthroplasty (TKA) in the same hospital, operated by the same surgeon between June 5th 2019 and March 31st 2021, using the same surgical technique described below. The setting was a philanthropic hospital that serves the national public health system in the Amazon region in Brazil.

We did not include patients with BMI over 40 kg/m^2^, previous personal or family history of venous thromboembolism, coagulation disorders, liver diseases, gastrointestinal or cerebral hemorrhage in the previous 3 months, allergy to enoxaparin, under chronic use of anticoagulants or with diagnosed malignant tumors. We set as exclusion criteria the failure to return for follow-up consultations, deaths not related to venous thromboembolism events or surgery complications during the first 3 months.

### Routine surgical procedure and preoperative and postoperative care

Regardless of allocation, all patients included in this trial underwent the same TKA technique during the morning. It started with a neuraxial block by spinal anesthesia (with 15 mg of 0.5% hyperbaric bupivacaine and 0.1 mg of morphine) and cemented primary TKA via a transquadricipital medial parapatellar approach with patellar eversion. All patients received the same type of prosthesis with posterior cruciate ligament substitution: AKS PS (Baumer, Mogi Mirim, São Paulo, Brazil) without patella replacement. We used a 3.2 mm 2-way suction drain for all patients, removing it 48 hours after surgery. We applied a pneumatic tourniquet device to the thigh of all patients. We adjusted pressure to 150 mmHg above the systolic pressure, from the start of the surgery until the cementation of the prosthesis.

Patients received cefuroxime sodium (1.5 g every 12 hours for 24 hours) as antibiotic prophylaxis, starting at anesthetic induction. They also received intravenous tranexamic acid, administered at a 20 mg/kg dose, half during the anesthetic induction (10 mg/kg) and the other half (10 mg/kg) 15 minutes before the tourniquet was released. At the end of the surgery, the patient underwent an anesthetic block of the saphenous nerve in the adductor canal of the thigh guided by ultrasound to control pain and allow early walking. For this block, we used 0.5% bupivacaine with a vasoconstrictor (15 ml) plus distilled water (15 ml), making a volume of 30 ml, with a final concentration of bupivacaine of 0.25%. We did not perform local anesthetic infiltration of the capsule or soft tissues around the knee. This protocol was the same for all patients regardless of allocation.

The hospital’s physical therapy team introduced ambulation and range of motion (ROM) exercises about 6 h after surgery. All team members proposed the same gait training for the patients in this study (also regardless of allocation): walking with the aid of a walker, for an average of 10 minutes on the first day and increasing the following morning gradually according to the patient’s tolerance of pain. Ambulation exercises would not be performed if the patient had persistent postural hypotension or voluntary refusal only. Patients received blood transfusion if they had a Hb level of less than 8 g/dl or values between 8 and 9 g/dl if accompanied by clinical symptoms of anemia.

Hospital discharge was planned for the third postoperative day, provided that the patient could walk with support and was in good clinical condition. They received guidance from the physiotherapy team at discharge regarding specific exercises to gain ROM and gait training.

We asked all patients to return to the hospital outpatient ward for a follow-up on edema and general clinical condition on the 10th, 45th and 90th days after surgery. We gave them guidance about signs and symptoms, such as calf stiffness, pain and discoloration of the leg (symptoms of deep vein thrombosis, DVT) or in case of shortness of breath, chest pain and cough (pulmonary embolism, EP). We asked them to telephone the team if they had any symptoms and to come to the emergency room when necessary.

### Thromboprophylaxis interventions under evaluation in this trial

Participants were randomly allocated to Group E, receiving enoxaparin (pharmacological thromboprophylaxis) and Group M (mechanical). The dose of enoxaparin given to Group E was 40 mg subcutaneously (equal dose for all patients and regardless of weight), started 12 h after the end of surgery. A daily dose was maintained until the 10th postoperative day.

**Fig. 1:**
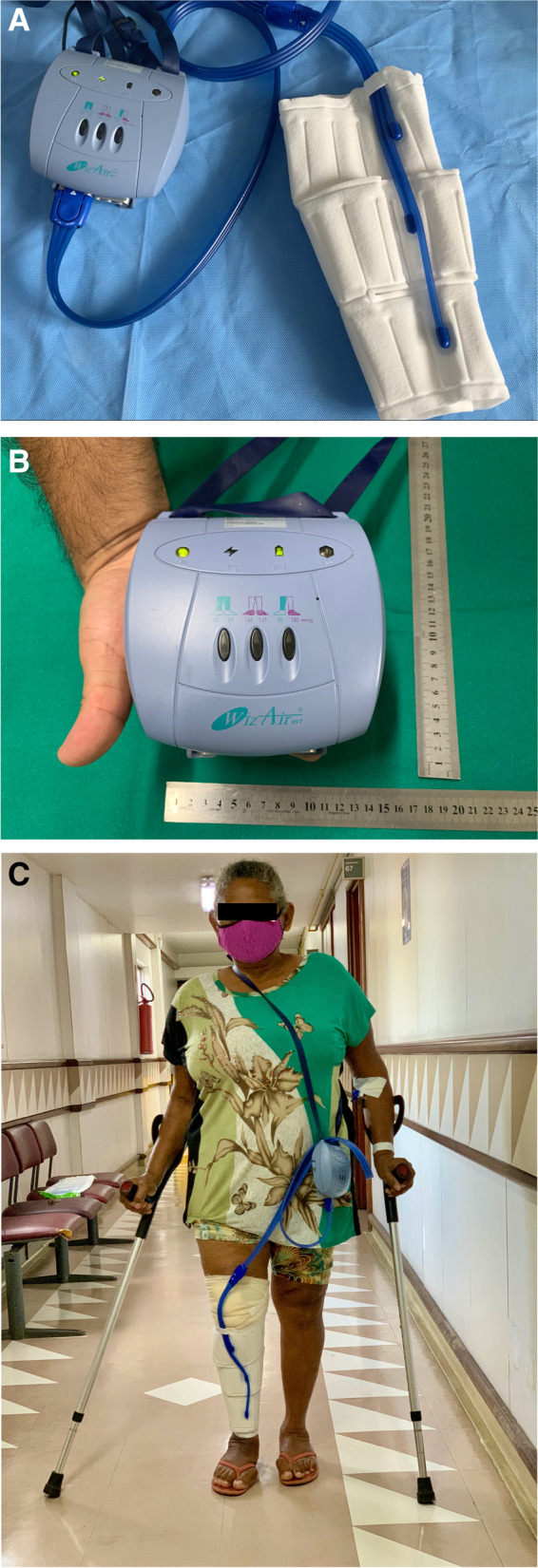
Photograph of the unilateral portable intermittent pneumatic compression device (IPCD) used in the trial (**A**); size of the pumping machine component (**B**) and device in use (**C**, with patient consent)

Group M received mechanical prophylaxis only, with a portable IPCD (WizAir, Medical Compression Systems, Or Akiva, Israel) for continuous use for 10 days, installed at the end of the surgery, right after the dressing, below the knee. The portable device allowed its use even while walking. A hose runs from this device to a sock that the patient wears on his/her leg. This sock has three independent air chambers, inflated by the hose in an ascending, progressive and intermittent manner (as shown in Fig. 1 and Suppl Video 1). The IPCD was placed only on the operated leg. The patient was instructed to wear it 24 hours a day, removing it only for showering. After hospital discharge, they were supposed to take it home and bring it back at the first hospital return visit 10 days later.

### Outcomes

The primary outcome, edema, was evaluated at the thigh, leg and ankle circumference in centimeters before surgery and on the third postoperative day. The standardized measurement was taken, always by the same physical therapist, at 10 cm proximally to the superior pole of the patella, for the thigh, 10 cm distal to the inferior pole of the patella, for the leg, and over the two malleoli for the ankle. This professional took the opportunity to examine the patient and register any skin changes, such as blisters or skin necrosis from edema. Surgeon also examined the skin during clinical visits.

Blood loss, the secondary outcome, was evaluated by the volume accumulated in the suction drain, in milliliters, and the hemoglobin (Hb, g/dl) and hematocrit (Ht, %) in the blood count taken in the morning before and again 48 hours after surgery. The nurse team registered the drained volume in the medical record as a routine. The hospital laboratory performed all blood counts. We also registered the time of tourniquet use.

We registered the following adverse events for this study: the need for blood transfusion and volume transfused; bleeding events and complications, according to the recommendation by ACCP, classified as fatal bleeding or hemorrhage requiring reoperation; infection and hospital readmission after discharge.

### Sample size calculation

The sample size calculation for this clinical study was based on the primary outcome, edema, indicated by the leg circumference measurement. A pilot study was carried out with the first 20 cases evaluated, in which the measurement change (post – preoperative) between the groups was observed. Knowing that the variability in leg circumference was 1.49 cm in this pilot study with the first 20 cases evaluated (standard deviation, SD, 1.49 cm), we calculated, assuming a mean difference of at least 0.90 cm, with 80% power and 95% confidence, that the sample needed would be 44 patients in each group. We opted for a larger sample of 150 patients (75 in each group), anticipating possible losses.

### Randomization, allocation concealment and blinding

The randomization was performed by an independent statistical professional. The statistician initially generated a simple random number sequence, without groups, using the Bio Estat program, version 5.3, with 150 numbers and their respective groups (M or E). With the patient allocation order in hand, he prepared 150 opaque envelopes with the sequential numbering written on the outside and the allocation (M or E) on a paper inside. Every time a patient was admitted to the surgical ward for the operation, a nurse in the operating room had to open each envelope, respecting the sequence number. The surgeon (principal researcher) was only then informed about the allocation. The allocation had to be revealed at this point so that the surgeon could immediately request the hospital pharmacy to send the IPCD to the operating room so that it could be applied immediately at the end of the surgery.

The physical therapist measuring the limb circumference for edema evaluation (primary outcome) could not be blinded at the measurement after surgery, as the IPCD is visible and was not removed. Patients could not be blinded either for the same reason.

### Statistical analysis

Categorical variables were described according to groups using absolute and relative frequencies, and the association with groups was verified using chi-square tests. Quantitative measures were described using summary measures (mean, standard deviation, median, minimum and maximum) and compared between groups using the Student’s t-test, as the Kolmogorov-Smirnov test indicated normal distribution.

Tourniquet time and bleeding were compared between groups, controlling for characteristics that showed differences in the preoperative period using general linear models (adjusted for age).

All measurements that were performed at two moments (preoperatively and after 48 hours, in the case of bleeding, and on the third day, in the case of edema) were described according to groups and moments and compared using equations of generalized estimation (EEG) with normal distribution and identity link function, assuming a first-order autoregressive correlation matrix between moments. The analyzes were followed by multiple Bonferroni comparisons to verify between which groups and moments the differences occurred. Contrasts were created to identify the differences between the pre and postoperative differences of the groups.

To perform the analyses, we used the IBM-SPSS for Windows version 20.0 software and, for data tabulation, the Microsoft Excel 2003 software. The tests were performed considering a significance level of 5%.

## Results

### Baseline evaluation and composition of the groups

**Fig. 2:**
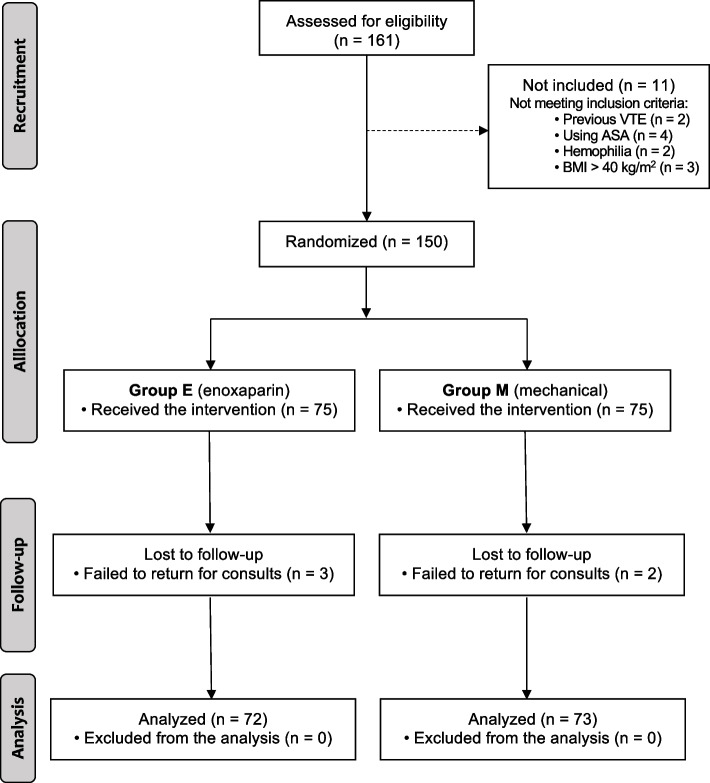
Flowchart of patients’ inclusions and exclusions

In the study period, 161 patients were admitted to the hospital for TKA, and 11 were excluded for the reasons described in Fig. 2, with 150 finally randomized. We lost the follow-up of 3 patients in Group E, and 2 in Group M. We could confirm by telephone that 4 of them were well and did not come for the follow-up consultations due to lack of transportation. We could not contact the last patient (Group E) or any family member.

Table 1 shows baseline clinical and demographic characteristics of operated patients. The mean tourniquet time use in Group E was 79.8 minutes and 76.8 for Group M (*p* = 0.188). Table 2 shows that Hb and Ht of Group M were 0.6 g/dl and 1.6% lower than in Group E (*p* = 0.002 and 0.009 respectively) at baseline.

**Table 1:** Baseline clinical and demographic characteristics of the operated patients (* Student’s t test; ** Chi-square test)

Variable	Group		Total	***p***
	Enoxaparin (*n* = 75)	Mechanical (*n* = 75)	(***n*** = 150)	
**Gender, n (%)**				0,379**
Female	49 (65,3)	54 (72)	103 (68,7)	
Male	26 (34,7)	21 (28)	47 (31,3)	
**Side, n (%)**				0,870**
Right	41 (54,7)	40 (53,3)	81 (54)	
Left	34 (45,3)	35 (46,7)	69 (46)	
**Age (years)**				0.098*
mean ± standard deviation	67,1 ± 8,6	64,8 ± 8,2	65,9 ± 8,4	
median (minimum; maximum)	67 (50; 87)	64 (48; 88)	66 (48; 88)	
**Height (m)**				0.557*
mean ± standard deviation	1,56 ± 0,08	1,55 ± 0,09	1,56 ± 0,08	
median (minimum; maximum)	1,54 (1,4; 1,8)	1,53 (1,4; 1,8)	1,54 (1,4; 1,8)	
**Weight (Kg)**				0.743*
mean ± standard deviation	72,3 ± 10,4	72,9 ± 11,9	72,6 ± 11,1	
median (minimum; maximum)	73 (51; 100)	72 (47; 115)	72 (47; 115)	
**BMI (Kg/m**^**2**^**)**				0.348*
mean ± standard deviation	29,7 ± 3,5	30,3 ± 4,7	30 ± 4,2	
median (minimum; maximum)	29,2 (22,7; 38,7)	29,2 (21,2; 44,9)	29,2 (21,2; 44,9)

**Table 2:** Serum hemoglobin and hematocrit values at baseline

Variable	Group		Total	***p***
	Enoxaparin (*n* = 75)	Mechanical (*n* = 75)	(***n*** = 150)	
**Hemoglobin (g/dl)**				**0.002**
mean ± standard deviation	13.2 ± 1.2	12.6 ± 1.1	12.9 ± 1.2	
median (minimum; maximum)	13.3 (10.3; 15.9)	12.6 (10.1; 15.5)	12.8 (10.1; 15.9)	
**Hematocrit (%)**				**0.009**
mean ± standard deviation	40.1 ± 3.8	38.5 ± 3.5	39.3 ± 3.7	
median (minimum; maximum)	40 (31; 50)	38 (31; 48)	39 (31; 50)	

### Does mechanical prophylaxis prevent edema?

The mean and median edema measurements increased statistically from the preoperative to the third postoperative day, regardless of the group (pMoment < 0.001; Table 3). The difference between groups was not significant (pGroup > 0.05). Considering the groups and moments together, the measures of edema in the ankle and leg were different (p Interaction < 0.05), with no difference for the thigh, for which they did not reach 2 cm in circumference. Leg circumference increased by approximately 2 cm for Group E and approximately 1.5 cm in Group M. The increase in ankle circumference, which in Group E was approximately 1.5 cm, was close to zero in Group M. The limb circumferences increased in both groups (*p* <  0.001), but edema in Group E was significantly higher for the leg and ankle (*p* <  0.002) compared to Group M.

**Table 3:** Edema, measured as the circumference of the thigh, leg and ankle according to groups and moments of evaluation. Generalized estimating equations (GEE) with normal distribution and identity link function

Variable	Group Enoxaparin (***n*** = 72)		Group Mechanical (***n*** = 73)		p _**Group**_	p _**Moment**_	p _**Interaction**_
	Pre-operative	Post-operative	Pre-operative	Post-operative			
**Thigh circumference (cm)**					0.543	**< 0.001**	0.436
mean ± standard deviation	46.6 ± 5.3	48 ± 5.3	47.2 ± 5.6	48.5 ± 5.5			
median (minimum; maximum)	46.5 (37; 58)	47.8 (37; 63)	47 (34; 60)	48.8 (35; 61)			
**Leg circumference (cm)**					0.614	**< 0.001**	**0.001**
mean ± standard deviation	34.2 ± 3.1	36.4 ± 3.6	34.4 ± 3.2	35.7 ± 3.3			
median (minimum; maximum)	34 (28; 42)	36 (30; 48.5)	34 (26; 41)	35.5 (28; 44)			
**Ankle circumference (cm)**					**0.009**	**< 0.001**	**< 0.001**
mean ± standard deviation	23.7 ± 1.9	25.2 ± 2.1	23.6 ± 1.7	23.8 ± 2			
median (minimum; maximum)	23.5 (20; 31)	25 (21; 33)	23.5 (20; 28)	23.5 (20; 30)			

p _Group:_ compares groups independently of the moment of evaluation; p _Moment_ compares the moments independently of groups; p _Interaction_ compares the behavior of the groups across timings

### Does mechanical prophylaxis prevent blood loss?

Hb and Ht levels differed between groups across the moments (pInteraction < 0.05; Table 4). The largest difference was in Group E (Table 5), but the values were not similar preoperatively either (Table 2). Hb and Ht decreased significantly in both groups (*p* <  0.001). But the reductions of Hb and Ht were significantly greater in Group E (*p* <  0.001). The volume of blood loss in the drain in the first 48 hours was greater in Group E (*p* <  0.001), regardless of patient’s age (Table 6).

**Table 4:** Serum hemoglobin and hematocrit values in the groups and moments. Generalized estimating equations (GEE) with normal distribution and identity link function

Variable	Group Enoxaparin (***n*** = 72)		Group Mechanical (***n*** = 73)		p _**Group**_	p _**Moment**_	p _**Interaction**_
	Pre-operative	Post-operative	Pre-operative	Post-operative			
**Hemoglobin (g/dl)**					0.090	**< 0.001**	**0.001**
mean ± standard deviation	13.2 ± 1.2	10.2 ± 1.3	12.6 ± 1.2	10.2 ± 1.3			
median (minimum; maximum)	13.4 (10.3; 15.9)	10.2 (6.7; 12.9)	12.5 (10.1; 15.5)	10.3 (7.8; 12.9)			
**Hematocrit (%)**					0.140	**< 0.001**	**0.001**
mean ± standard deviation	40.1 ± 3.8	30.3 ± 3.7	38.3 ± 3.4	30.6 ± 3.7			
median (minimum; maximum)	40 (31; 50)	30 (20; 39)	38 (31; 48)	31 (23; 38)			

p _Group:_ compares groups independently of the moment of evaluation; p _Moment_ compares the moments independently of groups; p _Interaction_ compares the behavior of the groups across timings

**Table 5:** Multiple comparisons of edema and indicators of blood loss values across moments: pre-operative (pre-op) or post-operative (post-op). Bonferroni’s multiplicity adjustment or ^a^contrast difference pre and post-op between groups

Variable	Comparison		Mean difference	Standard error	Degrees of freedom	***p***
Thigh circumference (cm)	Enoxaparin post-op - Enoxaparin pre-op		1.44	0.17	1	**< 0.001**
	Mechanical post-op - Mechanical pre-op		1.26	0.17	1	**< 0.001**
	Enoxaparin pre-op - Mechanical pre-op		−0.63	0.90	1	> 0.999
	Enoxaparin post-op - Mechanical post-op		− 0.45	0.90	1	> 0.999
	Enoxaparin (post-pre) - Mechanical (post-pre)^a^		0.19	0.28	143	0.509
Leg circumference (cm)	Enoxaparin post-op - Enoxaparin pre-op		2.14	0.17	1	**< 0.001**
	Mechanical post-op - Mechanical pre-op		1.32	0.17	1	**< 0.001**
	Enoxaparin pre-op - Mechanical pre-op		−0.14	0.55	1	> 0.999
	Enoxaparin post-op - Mechanical post-op		0.68	0.55	1	> 0.999
	Enoxaparin (post-pre) - Mechanical (post-pre)^a^		0.83	0.26	143	**0.002**
Ankle circumference (cm)	Enoxaparin post-op - Enoxaparin pre-op		1.53	0.12	1	**< 0.001**
	Mechanical post-op - Mechanical pre-op		0.21	0.12	1	0.447
	Enoxaparin pre-op - Mechanical pre-op		0.16	0.32	1	> 0.999
	Enoxaparin post-op - Mechanical post-op		1.47	0.32	1	**< 0.001**
	Enoxaparin (post-pre) - Mechanical (post-pre)^a^		1.32	0.17	143	**< 0.001**
Hemoglobin (g/dl)	Enoxaparin pre-op - Enoxaparin post-op		3.04	0.13	1	**< 0.001**
	Mechanical pre-op - Mechanical post-op		2.41	0.13	1	**< 0.001**
	Enoxaparin pre-op - Mechanical pre-op		0.62	0.20	1	**0.013**
	Enoxaparin post-op - Mechanical post-op		−0.01	0.20	1	> 0.999
	Enoxaparin (pre-post) - Mechanical (pre-post)^a^		0.63	0.18	143	**0.001**
Hematocrit (%)	Enoxaparin pre-op - Enoxaparin post-op		9.74	0.42	1	**< 0.001**
	Mechanical pre-op - Mechanical post-op		7.75	0.42	1	**< 0.001**
	Enoxaparin pre-op - Mechanical pre-op		1.78	0.61	1	**0.021**
	Enoxaparin post-op - Mechanical post-op		−0.21	0.61	1	> 0.999
	Enoxaparin (pre-post) - Mechanical (pre-post)^a^		1.99	0.60	143	**0.001**

**Table 6:** Blood volume collected in the drain over 48 hours adjusted for age (Student’s t test)

Variable	Group		Total	***p***
	Enoxaparin (*n* = 72)	Mechanical (*n* = 73)	(***n*** = 145)	
**Drain volume (mL)**				
mean ± standard deviation	566.1 ± 174.5	420.8 ± 142.5	493 ± 174.6	**< 0.001**
median (minimum; maximum)	565 (160; 1000)	400 (150; 900)	480 (150; 1000)	

### Is mechanical prophylaxis safe?

Four patients received a blood transfusion on the third postoperative day: three in Group E. No patient experienced fatal or severe bleeding. A 67-year-old patient in Group M had a suspicion of DVT, with marked swelling in the leg. She returned to the emergency room 20 days after surgery. Doppler ultrasound ruled out DVT, and lymphedema was diagnosed. No patient had symptomatic PE.

A 61-year-old patient in group E had an acute infection on the 12th postoperative day. In the hospital’s emergency room, a puncture with culture was positive for *Enterococcus faecalis*. He was reoperated with debridement and polyethylene exchange, remained hospitalized for 2 weeks with intravenous antibiotic therapy and, after discharge, had oral antibiotic therapy for another 4 weeks, with resolution. Another patient in Group E, 59 years old, had hemarthrosis, punctured on the 13th postoperative day (44 ml). The punctured blood culture was negative, but empiric oral antibiotic was still prescribed (ciprofloxacin, 500 mg, orally every 12 hours) for 14 days, with good evolution.

A 53-year-old diabetic and hypertensive patient from group M had a stroke on the seventh postoperative day. She was treated at a referral hospital where her family took her. On her last return to our center, at 90 days, she had mild hemiparesis and difficulty walking.

Four participants (2.6%), three from Group M and one from Group E, did not walk in the first 24 hours of surgery due to complaints of malaise and asthenia.

## Discussion

### Questions/purposes

Unilateral mechanical thromboprophylaxis after TKA safely resulted in less edema in the leg and ankle and blood loss than the pharmacological alternative in this randomized trial. IPCD was used continuously and allowed patients to walk.

### Background and rationale

The latest American Academy of Orthopaedic Surgeons (AAOS) guideline recommends mechanical and/or pharmacological prophylaxis for patients with no prior history of VTE, no bleeding disorder, and no liver disease [2]. ACCP and other guidelines [2, 13, 14] recommend mechanical prophylaxis for at least 18 hours a day with a portable IPCD allowing walking while recording patient’s adherence [1]. Although edema impairs rehabilitation of patients having TKA [3, 4], we could not find studies comparing a portable IPCD thromboprophylaxis with enoxaparin for this outcome. The few studies on edema [22–24] used plantar compression pumps associated with anticoagulants compared with a control group using the same anticoagulant only. These studies found a significant reduction of lower limb edema. We did not observe the edema reduction on the thigh as they did, possibly because we measured the limb circumference too early on the third postoperative day.

Among studies on mechanical prophylaxis, this is the first to use the portable IPCD unilaterally, on the operated leg only. There is no consensus on whether to use the device in one or both legs [15]. When the patient needs to stay in bed for other reasons, its use on both limbs seems reasonable. However, we encouraged mobility in both legs as soon as the effect of spinal anesthesia wore off. Also, most started walking in the first 6 h after surgery. In TKA, walking in the first 24 hours after surgery is associated with reduced DVT occurrence [25, 26]. It was not, however, the scope of this research to evaluate the effectiveness of the two methods on the occurrence of VTE: given the very low incidence of symptomatic VTE in TKA, and the already proven effectiveness of several methods in VTE prevention, it did not make sense to use VTE as a primary outcome, as this would require a very large number of patients, more characteristic of multicenter studies. Instead, we chose to study edema and blood loss, that are early events in the clinical pathway of operated patients, and with potential impact on their quality of life.

The incidence of DVT after arthroplasty is lower in the contralateral leg (between 3,2 and 5.5%) [27–29], which supports the use of the compression on the operated side only. Using the IPCD on one leg only naturally impacts on costs too, and comfort for the patient, two outcomes to be further studied — and important in cases where both interventions are equally effective and safe. One needs to consider, also, that those studies on DVT in the contralateral leg screened all patients [27–29], and not only the symptomatic ones — as recommended by ACCP [1] and AAOS [2]. The majority of patients with DVT are asymptomatic, though [11, 30] and using anticoagulant medication for these patients would only increase the risk of bleeding, infection and operative wound complications [8].

Besides the lower limb edema, we also observed less blood loss in the group using IPCD. The results in the literature of IPCD on blood loss are controversial and different when portable and non-portable devices are used [8–12]. The reason why IPCD can result in less, rather than more blood loss is not known [8–12] and should be further investigated. We compared the unilateral IPCD with enoxaparin, and not with aspirin — a drug with increasing popularity in the last years. The most recent international consensus on VTE prophylaxis in knee surgery, published after the results analysis for this trial, recommends aspirin [30]. However, it is not yet a global trend, and especially it was not the preference of 87% of Brazilian orthopaedic surgeons, who preferred enoxaparin according to a national survey conducted in 2021 [31]. This is why we decided to compare the IPCD with enoxaparin and not aspirin.

The AAOS guideline recommends that the prophylaxis be based on a discussion with the patient, lasting for a minimum of 10 to 14 days [2]. We opted for providing it for 10 days based on this AAOS recommendation and also on the fact that we encouraged walking very early, only 6 h after surgery. This early start is also supported by other studies showing the positive effect of walking early on the reduction of venous thrombosis [25, 26]. The guidelines that suggest longer thromboprophylaxis are usually based on hip replacement, not knee surgery [1, 2]. Two systematic reviews and two randomized controlled trials could not conclude in favor of extending the prophylaxis in total knee replacement [32–35].

### Limitations

However, studying portable IPCDs present some challenges. One clear limitation of this trial was the inability to blind the patient and the staff for the intervention used. This is a limitation inherent to the chosen intervention, which could not be bypassed with a different trial design or with a biomechanical engineering solution for a sham treatment in our setting.

Another important limitation was that, when this trial was conducted, there was no IPCD available in Brazil that could register the time of use, as recommended by ACCP [1]. Therefore, we could not check patient adherence to the intervention at home. However, the device use in the hospital was monitored, and the edema measurements were taken in the morning of discharge, still in the hospital, reflecting the IPCD use and not any other confounding factors in patients’ homes. Future studies should take the measurements on the tenth postoperative day as well and verify specifically how the use of IPCD affects rehabilitation exercises and walking.

Thromboprophylaxis options after TKA are still a matter of discussion. Portable devices that can prevent DVT and PE without increasing blood loss or other risks are certainly welcome and should be further investigated. A unilateral IPCD has proven a good alternative to isolated pharmacological prevention in this trial.

## Conclusions

Exclusively mechanical prophylaxis after TKA with a portable IPCD on the operated leg only reduces leg and ankle swelling and postoperative blood loss when compared to exclusively pharmacological prophylaxis with enoxaparin.

## Supplementary Information

**Additional file 1.** Video showing the inflation of the portable intermittent pneumatic compression device used in this research.

## Data Availability

The anonymized original research data has been uploaded to OSF: https://osf.io/xt4mk/?view_only=bce780010aa54b7cab11130859dd301a

## References

[1] Falck-Ytter Y, Francis CW, Johanson NA, Curley C, Dahl OE, Schulman S (2012). Prevention of VTE in orthopedic surgery patients: antithrombotic therapy and prevention of thrombosis, 9th ed: American College of Chest Physicians Evidence-Based Clinical Practice Guidelines. Chest..

[2] Mont MA, Jacobs JJ, Boggio LN, Bozic KJ, Della Valle CJ, Goodman SB (2011). Preventing venous thromboembolic disease in patients undergoing elective hip and knee arthroplasty. J Am Acad Orthop Surg.

[3] Pua YH (2015). The time course of Knee swelling post Total Knee Arthroplasty and its associations with quadriceps strength and gait speed. J Arthroplast.

[4] Loyd BJ, Stackhouse S, Dayton M, Hogan C, Bade M, Stevens-Lapsley J (2019). The relationship between lower extremity swelling, quadriceps strength, and functional performance following total knee arthroplasty. Knee..

[5] Jiang T, Song K, Yao Y, Pan P, Jiang Q (2019). Perioperative allogenic blood transfusion increases the incidence of postoperative deep vein thrombosis in total knee and hip arthroplasty. J Orthop Surg Res.

[6] Bierbaum BE, Callaghan JJ, Galante JO, Rubash HE, Tooms RE, Welch RB (1999). An analysis of blood management in patients having a total hip or knee arthroplasty. J Bone Joint Surg Am.

[7] Acuna AJ, Grits D, Samuel LT, Emara AK, Kamath AF (2021). Perioperative blood transfusions are associated with a higher incidence of thromboembolic events after TKA: an analysis of 333,463 TKAs. Clin Orthop Relat Res.

[8] Arsoy D, Giori NJ, Woolson ST (2018). Mobile compression reduces bleeding-related readmissions and wound complications after THA and TKA. Clin Orthop Relat Res.

[9] Chin PL, Amin MS, Yang KY, Yeo SJ, Lo NN (2009). Thromboembolic prophylaxis for total knee arthroplasty in Asian patients: a randomised controlled trial. J Orthop Surg (Hong Kong).

[10] Gelfer Y, Tavor H, Oron A, Peer A, Halperin N, Robinson D (2006). Deep vein thrombosis prevention in joint arthroplasties: continuous enhanced circulation therapy vs low molecular weight heparin. J Arthroplast.

[11] Kim KI, Kim DK, Song SJ, Hong SJ, Bae DK (2019). Pneumatic compression device does not show effective thromboprophylaxis following total knee arthroplasty in a low incidence population. Orthop Traumatol Surg Res.

[12] Sharfman ZT, Campbell JC, Mirocha JM, Spitzer AI (2016). Balancing Thromboprophylaxis and bleeding in Total joint Arthroplasty: impact of eliminating enoxaparin and Predonation and implementing pneumatic compression and Tranexamic acid. J Arthroplast.

[13] Afshari A, Fenger-Eriksen C, Monreal M, Verhamme P, Force EVGT (2018). European guidelines on perioperative venous thromboembolism prophylaxis: mechanical prophylaxis. Eur J Anaesthesiol.

[14] Anderson DR, Morgano GP, Bennett C, Dentali F, Francis CW, Garcia DA (2019). American Society of Hematology 2019 guidelines for management of venous thromboembolism: prevention of venous thromboembolism in surgical hospitalized patients. Blood Adv.

[15] Sadaghianloo N, Dardik A (2016). The efficacy of intermittent pneumatic compression in the prevention of lower extremity deep venous thrombosis. J Vasc Surg Venous Lymphat Disord.

[16] Odeh K, Doran J, Yu S, Bolz N, Bosco J, Iorio R (2016). Risk-stratified venous thromboembolism prophylaxis after Total joint Arthroplasty: aspirin and sequential pneumatic compression devices vs aggressive chemoprophylaxis. J Arthroplast.

[17] Nam D, Nunley RM, Johnson SR, Keeney JA, Barrack RL (2015). Mobile compression devices and aspirin for VTE prophylaxis following simultaneous bilateral total knee arthroplasty. J Arthroplast.

[18] Edwards JZ, Pulido PA, Ezzet KA, Copp SN, Walker RH, Colwell CW (2008). Portable compression device and low-molecular-weight heparin compared with low-molecular-weight heparin for thromboprophylaxis after total joint arthroplasty. J Arthroplast.

[19] Colwell CW, Froimson MI, Mont MA, Ritter MA, Trousdale RT, Buehler KC (2010). Thrombosis prevention after total hip arthroplasty: a prospective, randomized trial comparing a mobile compression device with low-molecular-weight heparin. J Bone Joint Surg Am.

[20] Colwell CW, Froimson MI, Anseth SD, Giori NJ, Hamilton WG, Barrack RL (2014). A mobile compression device for thrombosis prevention in hip and knee arthroplasty. J Bone Joint Surg Am.

[21] Schulz KF, Altman DG, Moher D, Group C (2011). CONSORT 2010 statement: updated guidelines for reporting parallel group randomised trials. Int J Surg.

[22] Windisch C, Kolb W, Kolb K, Grutzner P, Venbrocks R, Anders J (2011). Pneumatic compression with foot pumps facilitates early postoperative mobilisation in total knee arthroplasty. Int Orthop.

[23] Westrich GH, Sculco TP (1996). Prophylaxis against deep venous thrombosis after total knee arthroplasty. Pneumatic plantar compression and aspirin compared with aspirin alone. J Bone Joint Surg Am.

[24] Tamir L, Hendel D, Neyman C, Eshkenazi AU, Ben-Zvi Y, Zomer R (1999). Sequential foot compression reduces lower limb swelling and pain after total knee arthroplasty. J Arthroplast.

[25] Chandrasekaran S, Ariaretnam SK, Tsung J, Dickison D (2009). Early mobilization after total knee replacement reduces the incidence of deep venous thrombosis. ANZ J Surg.

[26] Pearse EO, Caldwell BF, Lockwood RJ, Hollard J (2007). Early mobilisation after conventional knee replacement may reduce the risk of postoperative venous thromboembolism. J Bone Joint Surg Br.

[27] Song K, Xu Z, Rong Z, Yang X, Yao Y, Shen Y (2016). The incidence of venous thromboembolism following total knee arthroplasty: a prospective study by using computed tomographic pulmonary angiography in combination with bilateral lower limb venography. Blood Coagul Fibrinolysis.

[28] Chang MJ, Song MK, Kyung MG, Shin JH, Chang CB, Kang SB (2018). Incidence of deep vein thrombosis before and after total knee arthroplasty without pharmacologic prophylaxis: a 128-row multidetector CT indirect venography study. BMC Musculoskelet Disord.

[29] Stulberg BN, Insall JN, Williams GW, Ghelman B (1984). Deep-vein thrombosis following total knee replacement. An analysis of six hundred and thirty-eight arthroplasties. J Bone Joint Surg Am.

[30] Hip I-V, Knee D (2022). Recommendations from the ICM-VTE: Hip & Knee. J Bone Joint Surg Am.

[31] Maradei-Pereira JAR, Barbosa MC, Newbery DFS, Torres MR, Kuhn A, Demange MK (2022). Preferências e práticas de ortopedistas brasileiros por técnicas de tromboprofilaxia na artroplastia total do joelho: Levantamento entre membros da Sociedade Brasileira de Cirurgia do Joelho (SBCJ). Rev Bras Ortop.

[32] Comp PC, Spiro TE, Friedman RJ, Whitsett TL, Johnson GJ, Gardiner GA (2001). Prolonged enoxaparin therapy to prevent venous thromboembolism after primary hip or knee replacement. Enoxaparin clinical trial group. J Bone Joint Surg Am.

[33] Forster R, Stewart M (2016). Anticoagulants (extended duration) for prevention of venous thromboembolism following total hip or knee replacement or hip fracture repair. Cochrane Database Syst Rev.

[34] Heit JA, Elliott CG, Trowbridge AA, Morrey BF, Gent M, Hirsh J (2000). Ardeparin sodium for extended out-of-hospital prophylaxis against venous thromboembolism after total hip or knee replacement. A randomized, double-blind, placebo-controlled trial. Ann Intern Med.

[35] Sobieraj DM, Lee S, Coleman CI, Tongbram V, Chen W, Colby J (2012). Prolonged versus standard-duration venous thromboprophylaxis in major orthopedic surgery: a systematic review. Ann Intern Med.

